# Diabetes mellitus in relation to colorectal tumor molecular subtypes: A pooled analysis of more than 9000 cases

**DOI:** 10.1002/ijc.34015

**Published:** 2022-04-22

**Authors:** Sophia Harlid, Bethany Van Guelpen, Conghui Qu, Björn Gylling, Elom K. Aglago, Efrat L. Amitay, Hermann Brenner, Daniel D. Buchanan, Peter T. Campbell, Yin Cao, Andrew T. Chan, Jenny Chang‐Claude, David A. Drew, Jane C. Figueiredo, Amy J. French, Steven Gallinger, Marios Giannakis, Graham G. Giles, Marc J. Gunter, Michael Hoffmeister, Li Hsu, Mark A. Jenkins, Yi Lin, Victor Moreno, Neil Murphy, Polly A. Newcomb, Christina C. Newton, Jonathan A. Nowak, Mireia Obón‐Santacana, Shuji Ogino, John D. Potter, Mingyang Song, Robert S. Steinfelder, Wei Sun, Stephen N. Thibodeau, Amanda E. Toland, Tomotaka Ugai, Caroline Y. Um, Michael O. Woods, Amanda I. Phipps, Tabitha Harrison, Ulrike Peters

**Affiliations:** ^1^ Department of Radiation Sciences, Oncology Unit Umeå University Umeå Sweden; ^2^ Wallenberg Centre for Molecular Medicine, Umeå University Umeå Sweden; ^3^ Public Health Sciences Division Fred Hutchinson Cancer Research Center Seattle Washington USA; ^4^ Department of Medical Biosciences, Pathology Unit Umeå University Umeå Sweden; ^5^ Nutrition and Metabolism Section International Agency for Research on Cancer, World Health Organization Lyon France; ^6^ Division of Clinical Epidemiology and Aging Research German Cancer Research Center (DKFZ) Heidelberg Germany; ^7^ Division of Preventive Oncology German Cancer Research Center (DKFZ) and National Center for Tumor Diseases (NCT) Heidelberg Germany; ^8^ German Cancer Consortium (DKTK), German Cancer Research Center (DKFZ) Heidelberg Germany; ^9^ Colorectal Oncogenomics Group, Department of Clinical Pathology The University of Melbourne Parkville Victoria Australia; ^10^ University of Melbourne Centre for Cancer Research, Victorian Comprehensive Cancer Centre Parkville Victoria Australia; ^11^ Genetic Medicine and Family Cancer Clinic, The Royal Melbourne Hospital Parkville Victoria Australia; ^12^ Department of Epidemiology & Population Health Albert Einstein College of Medicine Bronx New York USA; ^13^ Division of Public Health Sciences, Department of Surgery Washington University School of Medicine St Louis Missouri USA; ^14^ Alvin J. Siteman Cancer Center at Barnes‐Jewish Hospital and Washington University School of Medicine St. Louis Missouri USA; ^15^ Division of Gastroenterology, Department of Medicine Washington University School of Medicine St. Louis Missouri USA; ^16^ Division of Gastroenterology Massachusetts General Hospital and Harvard Medical School Boston Massachusetts USA; ^17^ Channing Division of Network Medicine, Brigham and Women's Hospital and Harvard Medical School Boston Massachusetts USA; ^18^ Clinical and Translational Epidemiology Unit, Massachusetts General Hospital and Harvard Medical School Boston Massachusetts USA; ^19^ Broad Institute of MIT and Harvard Cambridge Massachusetts USA; ^20^ Department of Epidemiology Harvard T.H. Chan School of Public Health, Harvard University Boston Massachusetts USA; ^21^ Department of Immunology and Infectious Diseases Harvard T.H. Chan School of Public Health, Harvard University Boston Massachusetts USA; ^22^ Division of Cancer Epidemiology German Cancer Research Center (DKFZ) Heidelberg Germany; ^23^ University Medical Centre Hamburg‐Eppendorf, University Cancer Centre Hamburg (UCCH) Hamburg Germany; ^24^ Department of Medicine Samuel Oschin Comprehensive Cancer Institute, Cedars‐Sinai Medical Center Los Angeles California USA; ^25^ Department of Preventive Medicine Keck School of Medicine, University of Southern California Los Angeles California USA; ^26^ Division of Laboratory Genetics, Department of Laboratory Medicine and Pathology Mayo Clinic Rochester Minnesota USA; ^27^ Lunenfeld Tanenbaum Research Institute, Mount Sinai Hospital, University of Toronto Toronto Ontario Canada; ^28^ Department of Medical Oncology Dana‐Farber Cancer Institute Boston Massachusetts USA; ^29^ Department of Medicine Brigham and Women's Hospital, Harvard Medical School Boston Massachusetts USA; ^30^ Cancer Epidemiology Division Cancer Council Victoria Melbourne Victoria Australia; ^31^ Centre for Epidemiology and Biostatistics, Melbourne School of Population and Global Health, The University of Melbourne Melbourne Victoria Australia; ^32^ Precision Medicine, School of Clinical Sciences at Monash Health, Monash University Clayton Victoria Australia; ^33^ Department of Biostatistics University of Washington Seattle Washington USA; ^34^ Oncology Data Analytics Program, Catalan Institute of Oncology (ICO), L'Hospitalet de Llobregat Barcelona Spain; ^35^ ONCOBEL Program, Bellvitge Biomedical Research Institute (IDIBELL), L'Hospitalet de Llobregat Barcelona Spain; ^36^ Consortium for Biomedical Research in Epidemiology and Public Health (CIBERESP) Madrid Spain; ^37^ Department of Clinical Sciences, Faculty of Medicine University of Barcelona Barcelona Spain; ^38^ School of Public Health, University of Washington Seattle Washington USA; ^39^ Department of Population Science American Cancer Society Atlanta Georgia USA; ^40^ Program in MPE Molecular Pathological Epidemiology, Department of Pathology Brigham and Women's Hospital and Harvard Medical School Boston Massachusetts USA; ^41^ Department of Oncologic Pathology Dana‐Farber Cancer Institute Boston Massachusetts USA; ^42^ Research Centre for Hauora and Health, Massey University Wellington New Zealand; ^43^ Department of Nutrition Harvard T.H. Chan School of Public Health, Harvard University Boston Massachusetts USA; ^44^ Departments of Cancer Biology and Genetics and Internal Medicine Comprehensive Cancer Center, The Ohio State University Columbus Ohio USA; ^45^ Memorial University of Newfoundland, Discipline of Genetics St. John's Canada; ^46^ Department of Epidemiology University of Washington Seattle Washington USA

**Keywords:** colorectal cancer, diabetes, subtype

## Abstract

Diabetes is an established risk factor for colorectal cancer. However, colorectal cancer is a heterogeneous disease and it is not well understood whether diabetes is more strongly associated with some tumor molecular subtypes than others. A better understanding of the association between diabetes and colorectal cancer according to molecular subtypes could provide important insights into the biology of this association. We used data on lifestyle and clinical characteristics from the Colorectal Cancer Family Registry (CCFR) and the Genetics and Epidemiology of Colorectal Cancer Consortium (GECCO), including 9756 colorectal cancer cases (with tumor marker data) and 9985 controls, to evaluate associations between reported diabetes and risk of colorectal cancer according to molecular subtypes. Tumor markers included *BRAF* and *KRAS* mutations, microsatellite instability and CpG island methylator phenotype. In the multinomial logistic regression model, comparing colorectal cancer cases to cancer‐free controls, diabetes was positively associated with colorectal cancer regardless of subtype. The highest OR estimate was found for *BRAF*‐mutated colorectal cancer, n = 1086 (OR_fully adj_: 1.67, 95% confidence intervals [CI]: 1.36‐2.05), with an attenuated association observed between diabetes and colorectal cancer without *BRAF*‐mutations, n = 7959 (OR_fully adj_: 1.33, 95% CI: 1.19‐1.48). In the case only analysis, *BRAF*‐mutation was differentially associated with diabetes (*P*_difference_ = .03). For the other markers, associations with diabetes were similar across tumor subtypes. In conclusion, our study confirms the established association between diabetes and colorectal cancer risk, and suggests that it particularly increases the risk of *BRAF*‐mutated tumors.

## Graphical abstract

What's new?

Diabetes is a well‐known risk factor for colorectal cancer, but colorectal cancer varies widely among patients. To better understand the association between diabetes and particular molecular subtypes of colorectal cancer, these authors analyzed data from 9,756 colorectal cancer cases and 9,985 controls. They found that diabetes appears to increase the risk of tumors with *BRAF* mutations, which generally have poorer outcomes. The large pooled dataset allowed detection of even small variations among subtypes, but the study also was not able to account for some potentially relevant factors, such as metformin use.

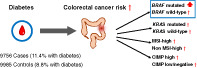

**Abbreviations**
BMI: body mass index
CCFR: Colorectal Cancer Family Registry
CIMP: CpG island methylator phenotype
CPS‐II: Cancer Prevention Study‐II
CRC: colorectal cancer
DACHS: The German Darmkrebs: Chancen der Verhütung durch Screening Study
EPIC: The European Prospective Investigation into Cancer and Nutrition Study
GECCO: Genetics and Epidemiology of Colorectal Cancer Consortium
HPFS: The Health Professionals Follow‐up Study
MCCS: The Melbourne Collaborative Cohort Study
MSI: microsatellite instability
NFCCR: The Newfoundland Colorectal Cancer Registry
NHS: The Nurses' Health Study
NSHDS: The Northern Sweden Health and Disease Study
OR: odds ratio

## 1 INTRODUCTION

Metabolic health, and excess body fat in particular, are involved in the development of colorectal cancer.^1^, ^2^, ^3^ Individuals with diabetes mellitus, especially those with type 2 diabetes, have an increased risk of developing colorectal cancer.^4^ This connection is likely independent of shared risk factors between diabetes and colorectal cancer.^5^ Instead, the association between diabetes and colorectal cancer may depend on other mechanisms such as alterations to the gut microbiome, increased inflammation in the gut, hyperinsulinemia in early stage type 2 diabetes and activation of cancer promoting pathways.^6^

Colorectal cancer is a heterogeneous disease, displaying considerable differences in molecular markers, which correlate with anatomical tumor location and other clinical and patient characteristics. For example, *BRAF*‐mutations are more common in proximal colon cancer, older patients and women and often co‐occur with high‐level microsatellite instability (MSI).^7^ This raises the question of whether the risk factors for colorectal cancer also vary by tumor molecular markers. Investigations into potentially variable associations of metabolic factors and molecular subtypes of colorectal cancer have been inconsistent, but some reports suggest a possible association between adiponectin and lower risk of *KRAS*‐mutated colorectal cancer.^8^, ^9^ Adiponectin is known to have anti‐inflammatory effects and has been suggested as a potential treatment for obesity and type 2 diabetes.^10^ Although studies have examined associations between body mass index (BMI) and different colorectal cancer phenotypes,^11^, ^12^ to our knowledge, no studies have investigated diabetes in relation to the risk of molecular subtypes of colorectal cancer, despite the fact that high BMI is one of the strongest known predictors of type 2 diabetes risk.^13^

The aim of the present study was to investigate self‐reported diabetes status in relation to molecular tumor traits in colorectal cancer (*BRAF* and *KRAS* mutations, MSI status and CpG island methylator phenotype [CIMP]). To achieve this, we used pooled data and tissue samples from the Colorectal Cancer Family Registry (CCFR) and the Genetics and Epidemiology of Colorectal Cancer Consortium (GECCO), comprising a total of 9756 colorectal cases, with tumor marker data, and 9985 colorectal cancer free controls, all with information about self‐reported diabetes status.

## 2 METHODS

### 2.1 Study participants

The study population consisted of individuals diagnosed with colorectal cancer and controls from observational studies within GECCO and the CCFR, with available tumor marker data and self‐reported information about diabetes status (not distinguishing between type 1 and type 2) (Supplementary Materials and Table S1). Specifically, 9756 CRC cases had characterization of MSI status, *BRAF* and *KRAS* mutations, and CIMP status.

Studies with MSI, *BRAF*, *KRAS* and CIMP data included the Seattle, Ontario, Australia and Mayo Clinic sites from the Colon Cancer Family Registry (CCFR), the Cancer Prevention Study‐II (CPS‐II), the German Darmkrebs: Chancen der Verhütung durch Screening Study (DACHS), the Swedish centers of the European Prospective Investigation into Cancer and Nutrition study (EPIC‐Sweden), the Health Professionals Follow‐up Study (HPFS), the Melbourne Collaborative Cohort Study (MCCS), the Newfoundland Colorectal Cancer Registry (NFCCR), the Nurses' Health Study (NHS) and the Northern Sweden Health and Disease Study (NSHDS, an EPIC partner but with no overlap with EPIC‐Sweden in our study).

### 2.2 Tumor marker data

#### 2.2.1 Collection and harmonization of MSI status CIMP status and *BRAF* and *KRAS* mutations

Data collection and harmonization of GECCO and CCFR tumor marker data have been described elsewhere.^14^, ^15^ Details on the analytical approach are described in the Supplementary Materials.

Briefly, MSI testing was primarily conducted using polymerase chain reaction (PCR) following the guidelines of the National Cancer Institute Bethesda Consensus Panel (CCFR, CPS‐II, MCCS, NHS)^16^ with ≥4 interpretable markers typically required to classify tumors (Table S2). DACHS used a mononucleotide panel of four markers. Tumors were classified as MSI‐high if at least 30% of the markers showed instability and non‐MSI‐high if less than 30% of the makers showed instability. Other studies used immunohistochemistry (NSHDS, EPIC‐Sweden and subsets of CCFR and MCCS).

CIMP status was determined using methylation analyses as described in the Supplementary Materials and Table S3. Briefly, the CCFR, CPS‐II, HPFS, MCCS, NSHDS, EPIC Sweden and NHS used MethyLight to determine CIMP status. CPS‐II, HPFS, NSHDS, EPIC Sweden and NHS used an 8‐gene panel; CCFR and MCCS used a 5‐gene panel. DACHS determined CIMP status using a different 5‐gene panel and methods described by Warth et al.^17^ We created two CIMP categories for this analysis: CIMP‐high and CIMP‐low/negative. In instances in which studies categorized tumors as CIMP‐high, CIMP‐low and CIMP‐negative, we combined CIMP‐low and CIMP‐negative into the CIMP‐low/negative category.

Studies assessed *BRAF* and *KRAS* mutations using PCR, sequencing and immunohistochemistry (Supplementary Materials). Most studies evaluated *BRAF* via c.1799T>A (V600E) mutations in exon 15 and *KRAS* via mutations in codons 12 and 13, although any mutation identified by one of the studies in *BRAF* and *KRAS* genes was included.

**FIGURE 1:**
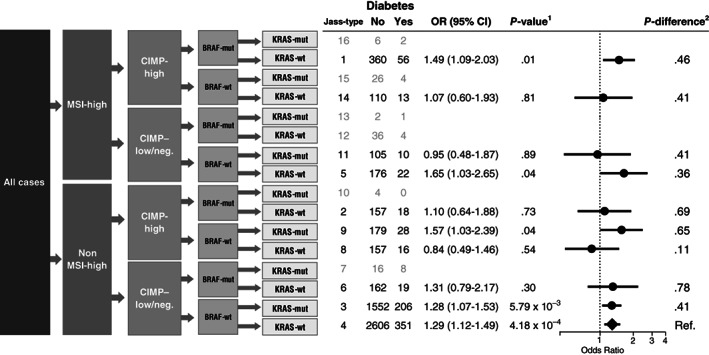
Case‐control associations between individuals reporting diabetes and individuals not reporting diabetes with risk of colorectal cancer subtypes defined by combined marker status. Error bars represent 95% confidence intervals. Adjusted for: study, sex, age at crc diagnosis, energy intake, family history, BMI, red meat, processed meat, vegetables, fruit, alcohol, smoking, exercise and aspirin/NSAID use. ^1^ *P*‐values were calculated using multinomial logistic regression, comparing colorectal cancer cases to cancer free controls separately for each defined Jass‐type with more than 50 cases included. ^2^ *P*_difference_ was calculated using multinomial logistic regression, comparing cases of each Jass‐type to all additional cases not belonging to that type

We further defined five combined colorectal tumor subtypes consistent with previously suggested classifications^18^, ^19^, ^20^: Type 1 (MSI‐high, CIMP‐high, BRAF‐mutated, KRAS‐wildtype), Type 2 (non MSI‐high, CIMP‐high, BRAF‐mutated, KRAS‐wildtype), Type 3 (non MSI‐high, CIMP‐low/negative, BRAF‐wildtype, KRAS‐mutated), Type 4 (non MSI‐high, CIMP‐low/negative, BRAF‐wildtype, KRAS‐wildtype) and Type 5 (MSI‐high, CIMP‐low/negative, BRAF‐wildtype, KRAS‐wildtype. Given our large sample size, we grouped additional marker combinations together into 11 additional types including: Type 6 (non MSI‐high, CIMP‐low/negative, *BRAF*‐mutated, *KRAS*‐wildtype), Type 8 (non MSI‐high, CIMP‐high, *BRAF*‐wildtype, *KRAS*‐mutated), Type 9 (MSI‐high, CIMP‐low/negative, *BRAF*‐wildtype, *KRAS*‐mutated), Type 11 (non MSI‐high, CIMP‐low/negative, *BRAF*‐wild type, *KRAS*‐mutated) and Type 14 (MSI‐high, CIMP‐high, *BRAF*‐wild type, *KRAS*‐wild type). Subtypes with fewer than 50 cases were dropped from analyses or were grouped together with the other marker combinations. These included: Type 7 (non MSI‐high, CIMP‐high, BRAF‐wildtype, KRAS‐wildtype), Type 10 (MSI‐high, CIMP‐high, BRAF‐wildtype, KRAS‐wildtype), Type 12 (MSI‐high, CIMP‐low/negative, BRAF‐mutated, KRAS‐wildtype), Type 13 (MSI‐high, CIMP‐low/negative, BRAF‐mutated, KRAS‐mutated), Type 15 (MSI‐high, CIMP‐high, BRAF‐wildtype, KRAS‐mutated) and Type 16 (MSI‐high, CIMP‐high, BRAF‐mutated, KRAS‐mutated). A summary of the combined marker classifications can be found in Figure 1 and Table S4.

### 2.3 Exposure data

Data collection and harmonization of GECCO and CCFR epidemiologic data have been described elsewhere.^14^, ^15^, ^21^ Briefly, demographic and environmental risk factor data were self‐reported at in‐person interviews or via structured self‐administered questionnaires. Data were collected at study entry, or 1 to 2 years prior to sample ascertainment. A multistep iterative data‐harmonization procedure was applied, reconciling each study's unique protocols and data collection instruments. Multiple quality‐control checks were performed, and outlying values of variables were truncated to the minimum or maximum value of an established range for each variable. Variables were combined into a single dataset with common definition, standardized coding and standardized permissible values.

Diabetes status was obtained through self‐reported answers to questions about diabetes diagnoses (summarized in Table S1) and includes, but does not distinguish between, both type 1 and type 2. We defined age at the time of a colorectal cancer diagnosis for cases and time of enrolment for controls. Missing covariate data were assumed to be missing at random, conditional on observed data and were imputed using mean imputation.

### 2.4 Statistical analyses

We used multinomial models to estimate odds ratios (OR) and 95% confidence intervals (CIs) for the association between diabetes and the risk of each molecular tumor marker among colorectal cancer cases, defined as MSI‐high vs non‐MSI‐high, CIMP‐high vs low/negative and *BRAF* or *KRAS* mutated vs nonmutated. To test for differences related to subtype within the case‐only analysis we used unconditional logistic regression. In the combined marker analysis, Type 4 (non‐MSI‐high, CIMP‐low/negative, *BRAF*‐wildtype, *KRAS*‐wildtype) was used as a reference group in the case‐only analysis, whereas in the polytomous analysis, cancer‐free controls were used as the reference group. Both analyses used multinomial logistic regression to compare each molecular pathological subtype to the reference group. We also used multinomial logistic regression to estimate the association between diabetes and risk of colorectal cancer stratified by tumor location (colon, rectum, proximal colon, distal colon) and sex (male and female), and compared case‐combinations of marker and tumor site with controls. For case‐only analyses, we compared marker combinations stratified by tumor site.

Minimally adjusted models (presented in the Supplementary Materials) included study, age and sex as covariates, and fully adjusted models additionally included energy intake, family history of colorectal cancer, BMI, red and processed meat consumption, vegetable consumption, fruit consumption, alcohol use, smoking status, exercise and aspirin/NSAID use. Variables were first selected based on their theoretical relevance as potential confounders of an association between diabetes and colorectal cancer risk. We then performed a forward direction selection of suitable covariates resulting in fiber intake being dropped from the analysis. For polytomous and case‐only analyses of the primary subtypes (MSI, *BRAF*, *KRAS* and CIMP‐status), we considered a two sided *P*‐value of <.05 to be significant (in both minimally and fully adjusted models). However, when testing associations between diabetes and combined marker subtypes, we used the alpha of 0.5% as recommended by Benjamin et al.^22^ All analyses were performed using R version 4.0.0 (R Foundation for Statistical Computing, Vienna).

## 3 RESULTS

The main characteristics of the study participants are described in Table 1. Individuals reporting a diabetes diagnosis were generally more likely than those not reporting diabetes to be male (*P* < .01) and to use aspirin regularly (*P* < .01), and less likely to have a reported family history of colorectal cancer, especially among colorectal cancer cases (*P* < .01). Participants with diabetes were also more often nondrinkers (*P* < .01), were more likely to exercise (*P* < .01), had a history of smoking (*P* < .01) and were more often obese (*P* < .01). These relationships were consistent among both colorectal cancer cases and controls.

**TABLE 1:** Participant characteristics

Characteristics	CRC cases		*P* ^a^	Controls		*P* ^a^
	Diabetes			Diabetes		
	No	Yes		No	Yes	
Total	8639	1117		9101	883	
Study, n (row %)			<.01			<.01
CCFR_Australia	713 (94.1)	45 (5.9)		175 (96.2)	7 (3.8)	
CCFR_Ontario	1073 (91.3)	102 (8.7)		1178 (90.1)	121 (9.9)	
CCFR_Seattle	1612 (87.5)	231 (12.5)		705 (93.0)	53 (7.0)	
CPSII	798 (93.0)	60 (7.0)		901 (93.0)	68 (7.0)	
DACHS	1879 (81.4)	430 (18.6)		2940 (86.0)	481 (14.0)	
EPIC_Sweden	142 (96.6)	5 (3.4)		384 (99.7)	1 (0.3)	
HPFS1	241 (96.0)	10 (4.0)		251 (98.8)	3 (1.2)	
HPFS2	354 (93.7)	24 (6.3)		192 (93.7)	13 (6.3)	
MCCS	467 (95.3)	23 (4.7)		658 (97.6)	16 (2.4)	
NFCCR	405 (78.9)	108 (21.1)		401 (86.1)	65 (13.9)	
NHS1	204 (95.8)	9 (4.2)		749 (97.5)	19 (2.5)	
NHS2	519 (89.5)	61 (10.5)		283 (90.1)	31 (9.9)	
NSHDS	232 (96.3)	9 (3.7)		284 (98.2)	5 (1.8)	
Age of CRC diagnosis			<.01			<.01
Mean (range)	62.57 (20‐96)	67.27 (27‐90)		65.75 (20‐102)	69.74 (27‐97)	
Sex, n (column %)			<.01			<.01
Female	4157 (48.1)	451 (40.4)		4505 (49.5)	318 (36)	
Male	4482 (51.9)	666 (59.6)		4596 (50.5)	565 (64)	
Family history of CRC, n (column%)			<.01			.38
No	6594 (76.3)	902 (80.8)		7656 (84.1)	780 (88.3)	
Yes	1571 (18.2)	169 (15.1)		947 (10.4)	87 (9.9)	
Aspirin, n (column %)			<.01			<.01
No	5949 (68.9)	668 (59.8)		5404 (59.4)	446 (50.5)	
Yes	1976 (22.9)	412 (36.9)		2736 (30.1)	419 (47.5)	
Dietary intake, mean (range)						
Energy intake, kcal/day	2046 (357‐4958)	2155 (310‐4959)	.01	1964 (379‐4958)	2043 (482‐4684)	.06
Redmeat, servings/day	0.72 (0‐8)	0.77 (0‐5)	.01	0.67 (0‐8)	0.75 (0‐5.2)	<.01
Process meat, servings/day	0.48 (0‐4)	0.66 (0‐2.9)	<.01	0.45 (0‐4)	0.64 (0‐2.5)	<.01
Vegetable, servings/day	2.21 (0‐20)	1.77 (0‐14)	<.01	2.24 (0‐18)	1.76 (0.03‐17)	<.01
Fruit, servings/day	1.6 (0‐20)	1.41 (0‐9)	<.01	1.69 (0‐20)	1.36 (0‐11)	<.01
Fiber, g/day	22.5 (3.2‐80)	23.4 (5.6‐70.4)	.11	22.7 (1.8‐80)	23.0 (3.8‐80)	.65
Alcohol intake, n (column %)			<.01			<.01
>28 g/day	1074 (12.4)	124 (11.1)		946 (10.4)	89 (10.1)	
1‐28 g/day	3854 (44.6)	412 (36.9)		4624 (50.8)	393 (44.5)	
Nondrinker	3135 (36.3)	539 (48.3)		3078 (33.8)	356 (40.3)	
Smoke ever, n (column %)			<.01			<.01
No	3597 (41.6)	419 (37.5)		4311 (47.4)	324 (36.7)	
Yes	4810 (55.7)	672 (60.2)		4547 (50)	533 (60.4)	
Exercise, n (column %)			<.01			<.01
No	332 (3.8)	25 (2.2)		427 (4.7)	20 (2.3)	
Yes	2642 (30.6)	471 (42.2)		3892 (42.8)	532 (60.2)	
BMI, n (%)			<.01			<.01
Normal	3096 (35.8)	157 (14.1)		3792 (41.7)	180 (20.4)	
Overweight	3613 (41.8)	459 (41.1)		3778 (41.5)	396 (44.8)	
Obese	1632 (18.9)	469 (42)		1307 (14.4)	287 (32.5)	
Stage, n (%)			.19			
Stage 1 or local	1746 (20.2)	219 (19.6)		—	—	
Stage 2/3 or regional	4735 (54.8)	687 (61.5)		—	—	
Stage 4 or distant	928 (10.7)	124 (11.1)		—	—	
Site, n (%)			.02			
Proximal	3173 (36.7)	460 (41.2)		—	—	
Distal	2525 (29.2)	298 (26.7)		—	—	
Rectal	2759 (31.9)	339 (30.3)		—	—	
*BRAF*, n (%)			.02		—	
Wildtype	7053 (81.6)	906 (81.1)		—	—	
Mutated	937 (10.8)	149 (13.3)		—	—	
*KRAS*, n (%)			.74		—	
Wildtype	4675 (54.1)	603 (54)		—	—	
Mutated	2315 (26.8)	306 (27.4)		—	—	
MSI, n (%)			.92		—	
Non MSI‐high	6843 (79.2)	877 (78.5)		—	—	
MSI‐high	1152 (13.3)	149 (13.3)		—	—	
CIMP, n (%)			.49		—	
Low/negative	5741 (66.5)	737 (66)		—	—	
High	1162 (13.5)	159 (14.2)		—	—	

^a^ *P*‐values from a *χ* ^2^ test for categorical variables and ANOVA for continuous variables.

In the case‐control analysis, we observed an association between diabetes and colorectal cancer (OR_all crc[fully adj.]_ = 1.34, 95% CI: 1.21‐1.48). This relationship was consistent across all molecular subtypes as shown in Table 2. However, it was stronger in cases with *BRAF*‐mutated tumors (OR_fully adj._ = 1.67, 95% CI: 1.36‐2.05) as compared to tumors without *BRAF* mutations (OR_fully adj._ = 1.33, 95% CI: 1.19‐1.48) (*P*_difference_ = .03) (Tables 2 and S5). For additional comparisons of subtype‐specific associations, differences were not significant.

**TABLE 2:** Associations between diabetes and risk of different molecular subtypes of colorectal cancer

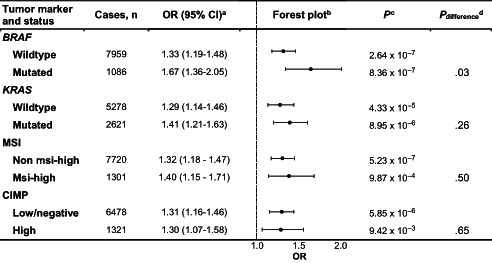

^a^ Adjusted for: study, sex, age at CRC diagnosis, energy intake, family history, BMI, red meat, processed meat, vegetables, fruit, alcohol, smoking, exercise and aspirin/NSAID use.

^b^ Error bars represent 95% confidence intervals.

^c^ Multinomial logistic regression was used to compare colorectal cancer cases to cancer free controls separately for each molecular pathological subtype (polytomous analysis, *P*).

^d^ Multinomial logistic regression was used to compare cases of each molecular pathological subtype to all additional cases not belonging to that subtype (case only analysis, *P*_difference_).

Analyses were then further stratified by tumor subsite and sex (Table S6).

We observed significant differences between the association of diabetes and colorectal cancer by *BRAF*‐mutation status for colon (*P*_difference_ = .04) and proximal colon cancers (*P*_difference_ = .02). The difference between *BRAF‐*mutated and wild‐type OR point estimates was also retained for rectal tumors (but not for distal tumors) and among both men and women, but without reaching significance (Table S6). However, the number of *BRAF*‐mutated tumors in the rectum was much lower than in the colon, which may explain why the observed difference in the association between diabetes and colorectal cancer risk by *BRAF*‐mutation status was not statistically significant. The association between diabetes and colorectal cancer risk did not differ by other tumor markers in any of the stratified analyses.

Results from analyses of combined marker subtypes are presented in Figure 1 and Table S7. Among the 10 subtype combinations tested, diabetes was statistically significantly associated with risk of five different types (1, 3‐5, and 9) in case‐control analyses, but only type 4 remained significant after adjustments for multiple comparisons. No significant differences in subtype‐specific associations were noted in case‐only analyses.

## 4 DISCUSSION

This large collaborative effort is the first study to investigate the impact of diabetes on subtypes of colorectal cancer, while also considering both distinct molecular markers and tumor subtypes based on marker combinations. Our primary finding was a statistically significant difference in the strength of the association between reported diabetes status and colorectal cancer by *BRAF* status, with a stronger association for *BRAF*‐mutated than nonmutated tumors. This was consistent in both our minimally and fully adjusted models. Reporting a diabetes diagnosis was more strongly associated with *BRAF*‐mutated tumors in both the proximal colon and rectum indicating that tumor location does not explain this result.

*BRAF* mutations, found in 8% to 12% of colorectal cancers, are generally associated with a poor prognosis and are more common in proximal tumors.^23^, ^24^, ^25^ BRAF is a serine‐threonine kinase activated by KRAS as part of the MAPK signaling cascade.^26^ Both *BRAF* and *KRAS* are oncogenes that are commonly mutated in colorectal cancer, and mutations in these genes are often considered mutually exclusive. Previous studies have found some evidence that medical drugs can differentially affect the risk of different molecular subtypes. For example, a study from 2013^27^ found that aspirin use (which has consistently been shown to decrease colorectal cancer risk^28^) seemed to specifically lower the risk of *BRAF*‐nonmutated colorectal cancer but not *BRAF*‐mutated colorectal cancer. More related to diabetes, a study from 2012^29^ found that metformin, an antidiabetic drug previously shown to have antitumor activity in several different cancers,^30^ did not affect *BRAF*‐mutated melanoma cells, but instead seemed to accelerate their growth in a xenograft mice model of melanoma. The authors suggested that the accelerated growth of the *BRAF*‐mutated tumors could be attributed to improved angiogenesis. Although this is in line with our own results, where individuals reporting diabetes had a higher incidence of *BRAF*‐mutated tumors compared to other molecular markers, later studies have been unable to replicate the findings.^31^ In addition, at least one study^32^ specifically examining the association between metformin and colon or colorectal cancer, but not by subtype, did not report any evidence of metformin acting differently depending on tumor site. It should also be noted that although metformin is commonly prescribed to individuals with type 2 diabetes mellitus, we lacked information about metformin use among our participants.

Colorectal cancer is known to often develop through a specific number of events along the so‐called conventional pathway, which is a multistep process initiated by mutations in *APC*, *KRAS* or *BRAF* genes. It is also well known that other important pathways exist, such as the serrated pathway and the alternate pathway, which have other characteristics as well as distinct risk factors.^33^ For example, the serrated pathway is associated with older age at onset, female sex and smoking and is also characterized primarily by *BRAF* mutations and CIMP‐high status.^34^, ^35^ Previous studies originating from the GECCO consortia have investigated whether other established risk factors for colorectal cancer are associated with specific molecular subtypes, sometimes supporting development through distinct pathways. One recent study focused on dietary factors did show some evidence of heterogeneity related to fruit and especially fiber intake.^15^ In polytomous analyses, higher fruit intake was associated with a decreased risk of developing *BRAF*‐mutated tumors. High fiber intake, on the other hand, was associated with decreased risk of non‐MSI‐high, CIMP‐low/negative, *BRAF*‐wildtype and *KRAS*‐wildtype subtypes, although none of these associations retained significance in case‐only analyses. In another study, it was found that smoking was strongly associated with subtype combinations that included CIMP‐high and MSI‐high tumors.^36^ As a result of these findings, the authors suggest that smoking might specifically increase the risk of tumors developing through the serrated pathway. Our study however, despite showing an increased risk of developing *BRAF*‐mutated tumors, did not find any evidence of diabetes resulting in any pathway specific development. Both previous studies and ours underline the importance of using large enough sample sets to be able to adequately assess patterns of associations that differ depending on molecular subtype.

Metabolic dysfunction, often defined as the presence of three or more of the criteria for metabolic syndrome (central obesity, hypertension, dysglycemia and dyslipidemia), is associated with an increased risk of developing both diabetes (type 2) and colorectal cancer. Several studies have aimed to assess the relationship between metabolic abnormalities (such as high BMI, hypertension and dysglycemia) and colorectal cancer, focusing on metabolic syndrome, inflammation and specific colorectal cancer subtypes.^37^, ^38^, ^39^ One of several plausible links between diabetes and colorectal cancer could be related to insulin resistance and hyperinsulinemia, a state of heightened insulin levels and a hallmark of untreated type 2 diabetes in its earlier natural history. High insulin levels have been linked to an increased risk of multiple cancers, including colorectal cancer.^40^ This may relate to its growth promoting effects as well as its ability to increase circulating IGF1 levels.^41^ Several studies have also identified a link between markers of heightened insulin levels (eg, C‐peptide) and colorectal cancer risk,^42^, ^43^ but without finding any evidence of heterogeneity.^44^ However, the most important metabolic factor likely to affect both diabetes and colorectal cancer risk is obesity and the relationship between BMI and colorectal cancer has also shown consistent directions of associations across studies, although just as for hyperglycemia, evidence of heterogeneity between subtypes has been somewhat inconsistent.^11^, ^45^, ^46^, ^47^, ^48^, ^49^, ^50^, ^51^ In the current study however, we find some evidence of diabetes increasing the risk of specifically *BRAF*‐mutated tumors, but taking into account previous studies, this subtype‐specific risk difference is probably not related to the shared metabolic risk factors connecting diabetes and colorectal cancer.

An important strength of our study is the ability to pool data from multiple observational studies with readily available information on diabetes status. This pooling of datasets enabled us to detect even modestly differential associations between diabetes status and risk of colorectal cancer by molecular subtypes. However, there are also limitations that have to be taken into account when interpreting the study results. First, we were not able to distinguish between type 1 and type 2 diabetes, or to take into account use of specific medications such as metformin. Time and duration of diabetes diagnosis was also lacking. These are all factors that can have important implications and result in misclassification bias. However, the size of our study, and the fact that type 2 diabetes makes up more than 90% of all diabetes cases,^52^ makes it less likely that these have substantially distorted our results. There may also be selection bias related to the cases included in the pooled analysis or to tissue availability potentially depending on tumor stage and size,^53^ a limitation that has been previously described.^15^ Finally, we did not apply any formal adjustment for multiple comparisons to our primary analyses. Although adding such adjustments would not affect the associations of risk, as they are all highly significant, the reported difference in OR estimates between *BRAF*‐mutated and nonmutated tumors would fall slightly below the significance threshold (accounting for tests of four different subtypes). This should be considered when interpreting the findings. It is worth noting that individuals reporting diabetes had a higher risk of all subtypes of colorectal cancer and the difference related to *BRAF*‐mutated tumors only affected the magnitude of the risk, but the direction remained the same.

In summary, our study confirms the established association between diabetes and colorectal cancer risk and suggests that it especially increases the risk of *BRAF*‐mutated tumors. Additional studies to confirm our finding, and possibly determine if the subtype specific association could be attributed to any particular component (such as metformin treatment), are needed before further conclusions can be drawn.

## CONFLICT OF INTEREST

Marios Giannakis reports potential financial conflicts of interest through research funding from Bristol‐Myers Squibb, Merck, Servier and Janssen unrelated to our study. Victor Moreno reports grants from Agency for Management of University and Research Grants (AGAUR) of the Catalan Government grant 2017SGR723; Instituto de Salud Carlos III grant PI17‐00092; Spanish Association Against Cancer (AECC) Scientific Foundation grant GCTRA18022MORE, Consortium for Biomedical Research in Epidemiology and Public Health (CIBERESP), action Genrisk. Jonathan Nowak reports prior research funding (for pilot projects) from NanoString and Illumina and ongoing research funding (for technology development) from Akoya Biosciences, unrelated to our study. Mireia Obón‐Santacana reports a post‐doctoral fellowship from the Spanish Association Against Cancer Scientific Foundation (AECC; POSTD037OBÓN). All other authors declare no conflicts of interest.

## DISCLAIMER

Where authors are identified as personnel of the International Agency for Research on Cancer / World Health Organization, the authors alone are responsible for the views expressed in this article and they do not necessarily represent the decisions, policy or views of the International Agency for Research on Cancer / World Health Organization.

## AUTHOR CONTRIBUTIONS

The work reported in the article has been performed by the authors, unless clearly specified in the text. Individual author contributions are as follows: Sophia Harlid: Conceptualization, investigation, project administration, visualization, writing‐original draft, writing‐review and editing; Bethany Van Guelpen: Conceptualization, resources, writing‐review and editing; Conghui Qu: Data curation, formal analysis, investigation, methodology, writing‐review and editing; Björn Gylling: Writing‐review and editing; Elom K. Aglago: Writing‐review and editing; Efrat L. Amitay: Writing‐review and editing; Hermann Brenner: Resources, writing ‐ review & editing; Daniel D. Buchanan: Resources, writing‐review and editing; Peter T. Campbell: Resources, writing‐review and editing; Yin Cao: Writing‐review and editing; Andrew T. Chan: Resources, writing‐review and editing; Jenny Chang‐Claude: Writing‐review and editing; David A. Drew: Writing‐review and editing; Jane C. Figueiredo: Writing‐review and editing; Amy J. French: Writing‐review and editing; Steven Gallinger: Resources, writing‐review and editing; Marios Giannakis: Writing‐review and editing; Graham G. Giles: Writing‐review and editing; Marc J. Gunter: Resources, writing‐review and editing; Michael Hoffmeister: Resources, writing‐review and editing; Li Hsu: Methodology, writing‐review and editing; Mark A. Jenkins: Resources, writing‐review and editing; Yi Lin: Data curation, software, writing‐review and editing; Victor Moreno: Resources, writing‐review and editing; Neil Murphy: Writing‐review and editing; Polly A. Newcomb: Resources, writing‐review and editing; Christina C. Newton: Writing‐review and editing; Jonathan A. Nowak: Writing‐review and editing; Mireia Obón‐Santacana: Writing‐review and editing; Shuji Ogino: Resources, writing‐review and editing; John D. Potter: Resources, writing‐review and editing; Mingyang Song: Writing‐review and editing; Robert S. Steinfelder: Data curation, writing‐review and editing; Wei Sun: Writing‐review and editing; Stephen N. Thibodeau: Resources, writing‐review and editing; Amanda E. Toland: Writing‐review and editing; Tomotaka Ugai: Writing‐review and editing; Caroline Y. Um: Writing‐review and editing; Michael O. Woods: Resources, writing‐review and editing; Amanda I. Phipps: Project administration, writing‐review and editing; Tabitha Harrison: Conceptualization, investigation, project administration, writing‐review and editing; Ulrike Peters: Conceptualization, supervision, funding acquisition, methodology, project administration, writing‐review and editing.

## ETHICS STATEMENT

All study participants had provided informed consent and included studies had obtained ethical approval from their respective research ethics committee or institutional review board.

## Supporting information


**Appendix S1**Supporting Information.

**Table S1**. Association between diabetes and risk of colorectal cancer stratified by tumor marker
**Table S2**. Association between diabetes and risk of individual molecular subtypes of colorectal cancer, stratified by tumor location and sex
**Table S3**. Minimally and fully adjusted case‐control associations between individuals with and without diabetes with risk of colorectal cancer subtypes defined by combined marker status. A two‐sided Wald test was used to calculate the *P*‐values from the case‐only analysis (*P*_difference_). Error bars represent 95% confidence intervals.

## Data Availability

Tumor marker and epidemiologic data is available upon request and permission. Please contact gro.hctuhderf@occeg to request the standardized proposal form. The principal investigators of each contributing study will evaluate and approve the proposal, and data access will be managed centrally. Further information is available from the corresponding authors upon request.
