# Supplementary material for: Diabetes mellitus in relation to colorectal tumor molecular subtypes: A pooled analysis of more than 9000 cases
Source: Int J Cancer. 2022 Apr 22;151(3):348–60. doi: 10.1002/ijc.34015 (PMC9251811; doi:10.1002/ijc.34015)
Supplement: Supplementary file 1 — Appendix S1Supporting Information. [file IJC-151-348-s002.pdf]

Supplemental materials

Diabetes mellitus in relation to colorectal tumor molecular subtypes - a pooled analysis of more than 9,000 cases

Sophia Harlid, Bethany Van Guelpen, Conghui Qu, Björn Gylling, Elom K Aglago, Efrat L Amitay, Hermann Brenner, Daniel D Buchanan, Peter T Campbell, Yin Cao, Andrew T Chan, Jenny Chang-Claude, David A Drew, Jane C Figueiredo, Amy J French, Steven Gallinger, Marios Giannakis, Graham G Giles, Marc J Gunter, Michael Hoffmeister, Li Hsu, Mark A Jenkins, Yi Lin, Victor Moreno, Neil Murphy, Polly A Newcomb, Christina C Newton, Jonathan A Nowak, Mireia Obón-Santacana, Shuji Ogino, John D Potter, Mingyang Song, Robert S Steinfelder, Wei Sun, Stephen N Thibodeau, Amanda E Toland, Tomotaka Ugai, Caroline Y Um, Michael O Woods, Amanda I Phipps,Tabitha Harrison, Ulrike Peters

Table of Contents: **Page**

Supplementary materials and methods:
Description of included studies 2
Harmonization of tumor marker data 5

Supplementary tables:
Table S1 (Participating studies and reported diabetes data) 7
Table S2 (Study-specific assessment of MSI status) 8
Table S3 (Study-specific assessment of CIMP status) 9
Table S4 (Combined colorectal tumor subtypes) 10
Table S5 (Diabetes and risk of colorectal cancer, **available in separate file**) -
Table S6 (Diabetes and risk of individual mol. subtypes, **available in separate file**) -
Table S7 (Jass-type specific associations, **available in separate file**) -

Supplementary references 11

Description of included studies

Colon Cancer Family Registry (CCFR)
The CCFR (www.coloncfr.org) is a National Cancer Institute-supported consortium consisting of six centers [1]. The CCFR includes data from approximately 42,500 total subjects (10,500 case probands and 26,900 unaffected and affected relatives, 4,280 unrelated population-based controls, and 920 spouses). The study recruited cases and unaffected controls (age 20 to 74 years) beginning in 1998. All participants self-completed a standardized questionnaire that included questions about established and suspected risk factors for colorectal cancer, including questions on medical history and medication use, reproductive history (for female participants), family history, physical activity, demographics, alcohol and tobacco use, and dietary factors. Participants from three of the six participating centers (Seattle-SCCFR, Australia-ACCFR, Ontario-OFCCR) were included in this study.

Cancer Prevention Study-II (CPS-II)
The CPS-II Nutrition cohort (established in 1992) is a prospective study of cancer incidence and mortality in the United States [2,3]. All participants filled out a self-administered questionnaire that included information on demographical, medical, dietary, and lifestyle factors. Biennial follow-up questionnaires have been sent out since 1997 in order to collect continuous information about current exposures and new cancer diagnoses. All reported cancers are verified through medical records, state cancer registry linkage, or death certificates. Controls were matched on race, gender, and age. The Emory University Institutional Review Board approves all aspects of the CPS-II Nutrition Cohort.

Darmkrebs: Chancen der Verhütung durch Screening (DACHS)
DACHS is a large German population-based case-control study started in 2003 in the Rhine-Neckar-Odenwald region (southwest region of Germany) [4,5]. The purpose of DACHS was to assess the potential of endoscopic screening for reduction of colorectal cancer risk and to investigate etiologic determinants of the disease, particularly lifestyle/environmental factors and genetic factors. Briefly, cases with a first diagnosis of invasive colorectal cancer (ICD-10 codes C18-C20) who were at least 30 years of age, German speaking, resident in the study region, and mentally and physically able to participate in a one-hour interview, were recruited by their treating physicians either in the hospital a few days after surgery, or by mail after hospital discharge. Cases were confirmed by histologic reports and hospital discharge letters following diagnosis of colorectal cancer. All hospitals treating colorectal cancer cancer patients in the study region participated. Communitybased controls were randomly selected from population registries, employing age frequency matching (5-year groups), sex, and county of residence. Controls without a history of colorectal cancer were contacted by mail and follow-up calls. During an in-person interview, data on demographics, medical history, family history of colorectal cancer, and various lifestyle factors were collected. Participants also donated blood and mouthwash samples.

European Prospective Investigation into Cancer (EPIC) - Sweden

EPIC is an on-going multicenter prospective cohort study designed to investigate the associations between diet, lifestyle, genetic and environmental factors and various types of cancer [6]. Briefly, 521,448 participants (~70% women) mostly aged 35 years or above were recruited between 1992 and 2000. Participants were recruited from 23 study centers in ten European countries. All study participants provided written informed consent, and ethical approval for the EPIC study was obtained from the review boards of IARC and local participating centers. The current study included participants from the northern Swedish EPIC-Umeå site, which is the Västerbotten Intervention Study (VIP). Colorectal cancer cases were identified by linkage with the Cancer Registry of Northern Sweden, which reports to the Swedish Cancer Registry, and were verified by a gastrointestinal pathologist. Controls were selected from the full cohort of individuals who were alive and free of cancer (except non-melanoma skin cancer) at the time of case diagnosis.

Health Professionals Follow-up Study (HPFS)

The HPFS was started in 1986 with the purpose of evaluating underlying etiologies of cardiovascular disease and cancer (8). It originally included 51,529 male health professionals currently residing in the United States who all completed a detailed questionnaire on health and diet. The all-male study was designed to complement the all-female Nurses' Health Study, which examines similar hypotheses. Colorectal cancer and other outcomes were reported by participants or next-of-kin and were followed up through review of the medical and pathology record by physicians. Overall, more than 97% of self-reported colorectal cancers were confirmed by medical record review. Information was abstracted on histology and primary anatomical location of the tumor. Follow-up evaluation has been excellent, with 94% of the men responding to date. Patients with available tumor molecular characterization were included in this study.

Melbourne Collaborative Cohort Study (MCCS)

The MCCS is a prospective study, run between 1990 and 1994, that recruited 41,514 healthy adult participants aged between 27 and 76 years (99% aged 40-69) from the Melbourne metropolitan area [7]. The goal of this study was to examine the role of lifestyle factors in the risk of cancer and heart disease. Incident cases of colorectal cancer were identified through linkage to population-based cancer registries in Australia. Cases included participants with a histopathological diagnosis of invasive colorectal adenocarcinoma diagnosed after baseline. Participants provided informed consent and sufficient FFPE material for somatic testing. Study protocols were approved by the Human Research Ethics Committee at the Cancer Council Victoria.

Newfoundland Familial Colorectal Cancer Registry (NFCCR)

The NFCCR is a case-control study that includes pathology confirmed colorectal cancer cases less than 75 years of age diagnosed between January 1999 and December 2003, as identified from the Newfoundland Cancer Registry. The Newfoundland Cancer Registry registers all cases of invasive cancer diagnosed among residents of the province of Newfoundland and Labrador in Canada. Consenting patients received a family history questionnaire and were asked to provide

a blood sample and to permit access to tumor tissue and medical records. If a patient was deceased, they sought participation of a close relative for the purposes of obtaining the family history and permission to access tissue blocks and medical records. Population-based controls were identified by random digit dialing from the residents of the province, and matched to the cases on sex and five-year age groups. Patients with available tumor molecular characterization were included in this study.

Nurses' Health Study (NHS)

The NHS cohort, initiated in 1976, originally included information on health related exposures from 121,700 married female registered nurses aged 30-55 [8]. Since 1976, follow-up questionnaires have been mailed every 2 years. Colorectal cancer and other outcomes were reported by participants or next-of-kin and followed up through review of the medical and pathology record by physicians. Overall, more than 97% of self-reported colorectal cancers were confirmed by medical-record review. Information was abstracted on histology and primary anatomical location of the tumor. The rate of follow-up evaluation has been high: as a proportion of the total possible follow-up time, follow-up evaluation has been more than 92%. Colorectal cancer cases were ascertained through June 1, 2008.

Northern Sweden Health and Disease Study (NSHDS)

The NSHDS is a population based study including residents of Västerbotten county in Northern Sweden. It includes more than 110,000 participants, of which approximately one third have repeated samples, from three population-based cohorts: the Västerbotten Intervention Project (VIP), the Northern Sweden WHO Monitoring of Trends and Cardiovascular Disease (MONICA) Study, and the local Mammography Screening Project (MSP). In the VIP cohort, which makes up approximately 85% of the NSHDS, aims to invite all residents of Västerbotten County to a health examination upon turning 30 (some years), 40, 50 and 60 years of age. It was established in 1985 and continues to recruit participants. In both the VIP and MONICA cohorts, extensive measured and self-reported health and lifestyle data were collected, whereas data in the MSP are more limited. Blood samples for research purposes are collected in all three cohorts. The NSHDS is a part of EPIC, and the selection of colorectal cases and controls were as described for EPIC-Sweden.

Harmonization of Colorectal Tumor Marker Data

Testing for microsatellite Instability (MSI), mutations in the BRAF gene, mutations in the KRAS gene, and CpG island methylator phenotype (CIMP) status was conducted by each study and according to individual study protocols. The harmonisation procedures have been previously described [9,10].

Microsatellite Instability (MSI) Status

Most studies used polymerase chain reaction (PCR) based assessment of microsatellite status, with the exception of NSHDS and EPIC-Sweden (13,14), which utilized immunohistochemical (IHC) detection of deficiency for mismatch repair (MMR) gene proteins MLH1, MSH2, MSH6, and PMS2 using standard procedures. Additionally, IHC was used for a subset of MCCS (15–17) and CCFR (1,18) samples. For classification using IHC, tumors lacking nuclear staining in tumor cells for at least one of these proteins were considered to have a positive MSI screening status and MSI negative screens were considered microsatellite stable (MSS). The specific markers assessed using PCR-based methods are summarized in Supplemental Table S2. To harmonize markers across all studies, we created two categories for downstream analyses, MSI-high and non MSI-high. For studies that categorized MSI status as MSI-high (MSI-H), MSI-low (MSI-L), and MSS, we collapsed MSI-L and MSS into the non MSI-high category.

Tumor classification was based on >4 interpretable markers for CCFR [1,11], NFCCR [12,13], MCCS [14], >5 interpretable markers for CPS-II (unless all four markers were unstable in which case the tumor was classified as MSI), and >7 interpretable markers for NHS and HPFS [15]. For these studies, tumors were classified as MSI-high (MSI-H) if 30% or more of the markers showed instability and non MSI-high if < 30% and > 0% showed instability, or if no marker exhibited instability.

DACHS [16] determined MSI status using a mononucleotide marker panel [17] that has high concordance with the National Cancer Institute Bethesda Consensus Panel [18].

BRAF and KRAS Mutation Status

Studies used PCR, sequencing, and IHC techniques to assess BRAF and KRAS mutations. The majority of studies evaluated the V600E mutation in BRAF exon 15 and KRAS mutations in codons 12 and 13, though a few evaluated additional loci. In analyses, we included any mutation identified by at least one study.

CCFR tested for the BRAF V600E mutation using a fluorescent allele-specific PCR (AS-PCR) assay [19] and used Sanger sequencing to assess mutations in KRAS codons 12 and 13 [20,21]. NFCCR tested for the BRAF V600E mutation using AS-PCR, followed by direct automatic sequencing to verify mutations [22], and did not evaluate KRAS mutations. MCCS used a fluorescent real-time AS-PCR assay [19] to test for the BRAF V600E mutation and a real-time PCR with high resolution melting (HRM) analysis followed by direct Sanger sequencing for positive cases to identify KRAS mutations in codons 12 and 13 [23]. CPS-II used PCR to assess BRAF V600E mutations and KRAS codon 12, 13, and 14 mutations.

DACHS [5] used both Sanger sequencing and IHC analysis of V600E expression to determine BRAF mutation status. For sequencing, they amplified exon 15 of BRAF using FideliTaq polymerase and sequenced using the BigDye Terminator v1.1 Cycle Sequencing Kit on an ABI 3500 Genetic Analyzer. DACHS determined KRAS mutation status by a single stranded conformational polymorphism technique (SSCP) or by Sanger sequencing, as reported previously [5]. NSHDS and EPIC Sweden [24] used real-time PCR using an allelic discrimination assay as described by Benlloch et. al. [25] to detect BRAF V600E mutations and BigDye v.3.1 sequencing to detect mutations in KRAS codons 12 and 13 [26].

HPFS and NHS performed PCR and pyrosequencing to identify BRAF codon 600 mutations [27–29]. HPFS and NHS used real-time PCR and pyrosequencing to identify KRAS mutations in codons 12, 13, 61, and 146 [27,30].

<u>CpG Island Methylator Phenotype Status</u>

Studies used gene promoter methylation analysis to determine CIMP status. The specific genes assessed in each study are shown in Supplemental Table S3. Similar to the harmonization of MSI status, we created two CIMP categories for downstream analyses, CIMP-high and CIMP-low/negative. In instances where studies categorized CIMP-high, CIMP-low, and CIMP-negative, we collapsed CIMP-low and CIMP-negative into the CIMP-low/negative category.

HPFS, NHS [28,31], CPS-II, NSHDS, EPIC Sweden [24,32], CCFR [33,34], MCCS [35] used the MethyLight [36] method to determine CIMP status. HPFS, NHS, CPS-II, NSHDS, and EPIC Sweden used a panel of eight genes, and CCFR and MCCS used a panel of five genes. The percent of methylated reference (PMR) value was calculated and, for CCFR, CPS-II, NSHDS, and EPIC Sweden a gene was considered positive for methylation when the PMR>10. HPFS and NHS used a PMR cutoff value of >4 for CDKN2A, MLH1, CACNA1G, NEUROG1, RUNX3, SOCS1, and a PMR of >6 for CRABP1 and IGF2. HPFS, NHS, CPS-II, and NSHDS classified tumors with ≥6 methylated markers as CIMP-high, 1-5 markers as CIMP-low, and no markers as CIMP-negative. CCFR and MCCS classified tumors with ≥3 methylated markers as CIMP-high and, otherwise, as CIMP-low/negative.

DACHS [37] determined CIMP status using a panel of five genes, and methods described by Warth et. al. [38]. They determined methylation status from the methylation-specific PCR based on the presence or absence of amplified product, and classified tumors with >3 methylated markers as CIMP-high, 1-2 markers as CIMP-low, and no markers as CIMP-negative.

Table S1. Description of participating studies

Study name	Abbreviation	Design	Country of origin	No. of CRC cases	No. of controls	Assessment of diabetes status
Colon Cancer Family Registry	CCFR_Australia	Case-control	Australia	758	182	Self-report: Ever diagnosed with diabetes?
Colon Cancer Family Registry	CCFR_Ontario	Case-control	Canada	1175	1299	Self-report: Ever diagnosed with diabetes?
Colon Cancer Family Registry	CCFR_Seattle	Case-control	United States	1843	758	Self-report: Ever diagnosed with diabetes?
Cancer Prevention Study II	CPSII	Cohort	United States	858	969	Self-report diabetes yes/no/unknown
Darmkrebs: Chancen der Verhütung durch Screenin	DACHS	Case–control	Germany	2309	3421	Self-report by patient, not asked for diagnosis by doctor.
European Prospective Investigation into Cancer_Sweden	EPIC_Sweden	Cohort	Sweden	147	385	Self-report: Ever diagnosed with diabetes by a doctor?
Health Professionals Follow-up Study 1	HPFS1	Cohort	United States	251	254	Self-report diabetes yes/no
Health Professionals Follow-up Study 2	HPFS2	Cohort	United States	378	205	Self-report diabetes yes/no
Melbourne Collaborative Cohort Study	MCCS	Cohort	Australia	490	674	Self-report: Ever diagnosed with diabetes by a doctor?
Newfoundland Familial Colorectal Cancer Registries	NFCCR	Case-control	Canada	513	466	Self-report: Has a doctor ever told you that you had diabetes?
Nurses' Health Study 1	NHS1	Cohort	United States	213	768	Self-report diabetes yes/no
Nurses' Health Study 2	NHS2	Cohort	United States	580	314	Self-report diabetes yes/no
Northern Sweden Health and Disease Study	NSHDS	Cohort	Sweden	241	289	Self-report: Ever diagnosed with diabetes?

Table S2. Summary of study specific assessment of microsatellite instability (MSI) status

Study	Markers*/ Proteins	Threshold for Interpretability	Definitions
CCFR	BAT25, BAT26, BAT40, BAT34C4, D5S346, D17S250, ACTC, D18S55, D10S197, MYCL	>4 interpretable markers	* MSI-H if >30% markers showed instability<br>* MSI-L if <30% and >0% showed instability<br>* MSS if no marker exhibited instability
CPSII	BAT25, BAT26, BAT40, BAT34C4,ACTC, D10S197, D17S250, D18S55, D5S346, MYCL	>5 interpretable markers (unless 4 markers were unstable)	* MSI-H if >30% markers showed instability<br>* MSI-L if <30% and >0% showed instability<br>* MSS if no marker exhibited instability
DACHS	BAT25, BAT26, CAT25	All 3 markers interpretable	* MSI-H if >1 marker showed instability<br>* MSS if 0 markers showed instability
EPIC_Sweden	MLH1, MSH2, MSH6, and PMS2	Immunohistochemistry	Immunohistochemical detection of deficiency for selected mismatch repair proteins was used to determine MSI status.
HPFS	BAT25, BAT26, BAT40, D18S55, D18S56, D18S67, D18S487, D2S123, D5S346, D17S250	>7 interpretable markers	* MSI-H if >30% markers showed instability<br>* MSI-L if <30% and >0% showed instability<br>* MSS if no marker exhibited instability
MCCS	BAT25, BAT26, BAT40, BAT34C4, D5S346, D17S250, ACTC, D18S55, D10S197, MYCL	>4 interpretable markers	* MSI-H if >30% markers showed instability<br>* MSI-L if <30% and >0% showed instability<br>* MSS if no marker exhibited instability
NFCCR	BAT-25, BAT-26, BAT-40, BAT-34C4, D5S346, D17S250, ACTC, D18S55, D10S197, MYCL	>4 interpretable markers	* MSI-H if >30% markers showed instability<br>* MSI-L if <30% and >0% showed instability<br>* MSS if no marker exhibited instability
NHS	BAT25, BAT26, BAT40, D18S55, D18S56, D18S67, D18S487, D2S123, D5S346, D17S250	>7 interpretable markers	* MSI-H if >30% markers showed instability<br>* MSI-L if <30% and >0% showed instability<br>* MSS if no marker exhibited instability
NSHDS	MLH1, MSH2, MSH6, and PMS2	Immunohistochemistry	Immunohistochemical detection of deficiency for selected mismatch repair proteins was used to determine MSI status.

*Includes mononucleotide, dinucleotide and other markers for MSI testing. A subset of CCFR and MCCS used immunohistochemical detection of deficiency for mismatch repair proteins.

Table S3. Summary of study specific assessment of CpG Island Methylation Phenotype (CIMP) status

Study	Panel genes	Marker positive definition	CIMP-high	CIMP-low/negative
CCFR	CACNA1G, IGF2, NEUROG1, RUNX3, and SOCS1	PMR > 10	≥3 methylated markers	≤2 methylated markers
CPSII	CDKN2A, MLH1, CACNA1G, NEUROG1, RUNX3, SOCS1, IGF2, CRABP1	PMR > 10	≥6 methylated markers	≤5 methylated markers
DACHS	MGMT, MLH1, MINT1, MINT2, MINT31	N/A	≥3 methylated markers	≤2 methylated markers
EPIC_Sweden	CDKN2A, MLH1, CACNA1G, NEUROG1, RUNX3, SOCS1, IGF2, CRABP1	PMR > 10	≥1 methylated markers	0 methylated markers
HPFS	CDKN2A, MLH1, CACNA1G, NEUROG1, RUNX3, SOCS1, IGF2, CRABP1	PMR > 4 for CDKN2A, MLH1, CACNA1G, NEUROG1, RUNX3, SOCS1. PMR > 6 for CRABP1, IGF2	≥6 methylated markers	≤5 methylated markers
MCCS	CACNA1G, IGF2, NEUROG1, RUNX3, and SOCS1	PMR > 10	≥3 methylated markers	≤2 methylated markers
NFCCR*	N/A	N/A	N/A	N/A
NHS	CDKN2A, MLH1, CACNA1G, NEUROG1, RUNX3, SOCS1, IGF2, CRABP1	PMR > 4 for CDKN2A, MLH1, CACNA1G, NEUROG1, RUNX3, SOCS1. PMR > 6 for CRABP1, IGF2	≥6 methylated markers	≤5 methylated markers
NSHDS	CDKN2A, MLH1, CACNA1G, NEUROG1, RUNX3, SOCS1, IGF2, CRABP1	PMR > 10	≥1 methylated markers	0 methylated markers

*CIMP status was not assessed in the NFCCR.

Table S4. Combined colorectal tumor subtypes

Type	No. of cases	MSI status	CIMP status	*BRAF* status	*KRAS* status
**Previously specified marker combinations [39–41]**					
**1**	416	MSI-high	High	*BRAF* mutated	*KRAS* wildtype
**2**	175	non MSI-high	High	*BRAF* mutated	*KRAS* wildtype
**3**	1758	non MSI-high	Low/Negative	*BRAF* wildtype	*KRAS* mutated
**4**	2957	non MSI-high	Low/Negative	*BRAF* wildtype	*KRAS* wildtype
**5**	198	MSI-high	Low/Negative	*BRAF* wildtype	*KRAS* wildtype
**Additional marker combinations**					
**6**	181	non MSI-high	Low/Negative	*BRAF* mutated	*KRAS* wildtype
**8**	173	non MSI-high	High	*BRAF* wildtype	*KRAS* mutated
**9**	207	MSI-high	Low/Negative	*BRAF* wildtype	*KRAS* mutated
**11**	115	non MSI-high	Low/Negative	*BRAF* wildtype	*KRAS* mutated
**14**	123	MSI-high	High	*BRAF* wildtype	*KRAS* wildtype
**Removed due to 50 or fewer cases**					
**7**	24	non MSI-high	High	*BRAF* wildtype	*KRAS* wildtype
**10**	4	MSI-high	High	*BRAF* wildtype	*KRAS* wildtype
**12**	40	MSI-high	Low/Negative	*BRAF* mutated	*KRAS* wildtype
**13**	3	MSI-high	Low/Negative	*BRAF* mutated	*KRAS* mutated
**15**	30	MSI-high	High	*BRAF* wildtype	*KRAS* mutated
**16**	8	MSI-high	High	*BRAF* mutated	*KRAS* mutated

Supplementary references

1. Newcomb PA, Baron J, Cotterchio M, Gallinger S, Grove J, Haile R, Hall D, Hopper JL, Jass J, Le Marchand L, Limburg P, Lindor N, et al. Colon Cancer Family Registry: an international resource for studies of the genetic epidemiology of colon cancer. *Cancer Epidemiol Biomarkers Prev* 2007;16:2331–43.
2. Calle EE, Rodriguez C, Jacobs EJ, Almon ML, Chao A, McCullough ML, Feigelson HS, Thun MJ. The American Cancer Society Cancer Prevention Study II Nutrition Cohort: rationale, study design, and baseline characteristics. *Cancer* 2002;94:2490–501.
3. Campbell PT, Deka A, Briggs P, Cicek M, Farris AB, Gaudet MM, Jacobs EJ, Newton CC, Patel AV, Teras LR, Thibodeau SN, Tillmans L, et al. Establishment of the cancer prevention study II nutrition cohort colorectal tissue repository. *Cancer Epidemiol Biomarkers Prev* 2014;23:2694–702.
4. Brenner H, Chang-Claude J, Jansen L, Knebel P, Stock C, Hoffmeister M. Reduced risk of colorectal cancer up to 10 years after screening, surveillance, or diagnostic colonoscopy. *Gastroenterology* 2014;146:709–17.
5. Jia M, Jansen L, Walter V, Tagscherer K, Roth W, Herpel E, Kloor M, Bläker H, Chang-Claude J, Brenner H, Hoffmeister M. No association of CpG island methylator phenotype and colorectal cancer survival: population-based study. *Br J Cancer* 2016;115:1359–66.
6. Riboli E, Hunt KJ, Slimani N, Ferrari P, Norat T, Fahey M, Charrondière UR, Hémon B, Casagrande C, Vignat J, Overvad K, Tjønneland A, et al. European Prospective Investigation into Cancer and Nutrition (EPIC): study populations and data collection. *Public Health Nutr* 2002;5:1113–24.
7. Giles GG, English DR. The Melbourne Collaborative Cohort Study. *IARC Sci Publ* 2002;156:69–70.
8. Belanger CF, Hennekens CH, Rosner B, Speizer FE. The nurses' health study. *Am J Nurs* 1978;78:1039–40.
9. Labadie JD, Harrison TA, Banbury B, Amtay EL, Bernd S, Brenner H, Buchanan DD, Campbell PT, Cao Y, Chan AT, Chang-Claude J, English D, et al. Postmenopausal hormone therapy and colorectal cancer risk by molecularly defined subtypes and tumor location. *JNCI Cancer Spectr* 2020;4:pkaa042.
10. Hidaka A, Harrison TA, Cao Y, Sakoda LC, Barfield R, Giannakis M, Song M, Phipps AI, Figueiredo JC, Zaidi SH, Toland AE, Amitay EL, et al. Intake of dietary fruit, vegetables, and fiber and risk of colorectal cancer according to molecular subtypes: A pooled analysis of 9 studies. *Cancer Res* 2020;80:4578–90.
11. Lindor NM, Burgart LJ, Leontovich O, Goldberg RM, Cunningham JM, Sargent DJ, Walsh-Vockley C, Petersen GM, Walsh MD, Leggett BA, Young JP, Barker MA, et al. Immunohistochemistry versus microsatellite instability testing in phenotyping colorectal tumors. *J Clin Oncol* 2002;20:1043–8.
12. Raptis S, Mrkonjic M, Green RC, Pethe VV, Monga N, Chan YM, Daftary D, Dicks E, Younghusband BH, Parfrey PS, Gallinger SS, McLaughlin JR, et al. MLH1 -93G>A promoter polymorphism and the risk of microsatellite-unstable colorectal cancer. *J Natl Cancer Inst* 2007;99:463–74.
13. Woods MO, Hyde AJ, Curtis FK, Stuckless S, Green JS, Pollett AF, Robb JD, Green RC, Croitoru ME, Careen A, Chaulk JAW, Jegathesan J, et al. High frequency of hereditary colorectal cancer in Newfoundland likely involves novel susceptibility genes. *Clin Cancer Res* 2005;11:6853–61.
14. Buchanan DD, Clendenning M, Rosty C, Eriksen SV, Walsh MD, Walters RJ, Thibodeau SN,

Stewart J, Preston S, Win AK, Flander L, Ouakrim DA, et al. Tumor testing to identify lynch syndrome in two Australian colorectal cancer cohorts. *J Gastroenterol Hepatol* 2017;32:427–38.

15. Ogino S, Brahmandam M, Cantor M, Namgyal C, Kawasaki T, Kirkner G, Meyerhardt JA, Loda M, Fuchs CS. Distinct molecular features of colorectal carcinoma with signet ring cell component and colorectal carcinoma with mucinous component. *Mod Pathol* 2006;19:59–68.

16. Hoffmeister M, Bläker H, Kloor M, Roth W, Toth C, Herpel E, Frank B, Schirmacher P, Chang-Claude J, Brenner H. Body mass index and microsatellite instability in colorectal cancer: a population-based study. *Cancer Epidemiol Biomarkers Prev* 2013;22:2303–11.

17. Findeisen P, Kloor M, Merx S, Sutter C, Woerner SM, Dostmann N, Benner A, Dondog B, Pawlita M, Dippold W, Wagner R, Gebert J, et al. T25 repeat in the 3’ untranslated region of the CASP2 gene: a sensitive and specific marker for microsatellite instability in colorectal cancer. *Cancer Res* 2005;65:8072–8.

18. Boland CR, Thibodeau SN, Hamilton SR, Sidransky D, Eshleman JR, Burt RW, Meltzer SJ, Rodriguez-Bigas MA, Fodde R, Ranzani GN, Srivastava S. A National Cancer Institute Workshop on Microsatellite Instability for cancer detection and familial predisposition: development of international criteria for the determination of microsatellite instability in colorectal cancer. *Cancer Res* 1998;58:5248–57.

19. Buchanan DD, Sweet K, Drini M, Jenkins MA, Win AK, English DR, Walsh MD, Clendenning M, McKeone DM, Walters RJ, Roberts A, Pearson S-A, et al. Risk factors for colorectal cancer in patients with multiple serrated polyps: a cross-sectional case series from genetics clinics. *PLoS ONE* 2010;5:e11636.

20. Alsop K, Mead L, Smith LD, Royce SG, Tesoriero AA, Young JP, Haydon A, Grubb G, Giles GG, Jenkins MA, Hopper JL, Southey MC. Low somatic K-ras mutation frequency in colorectal cancer diagnosed under the age of 45 years. *Eur J Cancer* 2006;42:1357–61.

21. Stewart CJR, Leung Y, Walsh MD, Walters RJ, Young JP, Buchanan DD. KRAS mutations in ovarian low-grade endometrioid adenocarcinoma: association with concurrent endometriosis. *Hum Pathol* 2012;43:1177–83.

22. Loughrey MB, Waring PM, Tan A, Trivett M, Kovalenko S, Beshay V, Young MA, McArthur G, Boussioutas A, Dobrovic A. Incorporation of somatic BRAF mutation testing into an algorithm for the investigation of hereditary non-polyposis colorectal cancer. *Fam Cancer* 2007;6:301–10.

23. Rosty C, Buchanan DD, Walsh MD, Pearson S-A, Pavluk E, Walters RJ, Clendenning M, Spring KJ, Jenkins MA, Win AK, Hopper JL, Sweet K, et al. Phenotype and polyp landscape in serrated polyposis syndrome: a series of 100 patients from genetics clinics. *Am J Surg Pathol* 2012;36:876–82.

24. Dahlin AM, Palmqvist R, Henriksson ML, Jacobsson M, Eklöf V, Rutegård J, Oberg A, Van Guelpen BR. The role of the CpG island methylator phenotype in colorectal cancer prognosis depends on microsatellite instability screening status. *Clin Cancer Res* 2010;16:1845–55.

25. Benlloch S, Payá A, Alenda C, Bessa X, Andreu M, Jover R, Castells A, Llor X, Aranda FI, Massutí B. Detection of BRAF V600E mutation in colorectal cancer: comparison of automatic sequencing and real-time chemistry methodology. *J Mol Diagn* 2006;8:540–3.

26. Myte R, Gylling B, Häggström J, Schneede J, Löfgren-Burström A, Huyghe JR, Hallmans G, Meyer K, Johansson I, Ueland PM, Palmqvist R, Van Guelpen B. One-carbon metabolism biomarkers and genetic variants in relation to colorectal cancer risk by KRAS and BRAF mutation status. *PLoS ONE* 2018;13:e0196233.

27. Ogino S, Kawasaki T, Brahmandam M, Yan L, Cantor M, Namgyal C, Mino-Kenudson M, Lauwers GY, Loda M, Fuchs CS. Sensitive sequencing method for KRAS mutation detection by Pyrosequencing. *J Mol Diagn* 2005;7:413–21.

28. Ogino S, Kawasaki T, Kirkner GJ, Loda M, Fuchs CS. CpG island methylator phenotype-low (CIMP-low) in colorectal cancer: possible associations with male sex and KRAS mutations. *J Mol Diagn* 2006;8:582–8.

29. Ogino S, Meyerhardt JA, Cantor M, Brahmandam M, Clark JW, Namgyal C, Kawasaki T, Kinsella K, Michelini AL, Enzinger PC, Kulke MH, Ryan DP, et al. Molecular alterations in tumors and response to combination chemotherapy with gefitinib for advanced colorectal cancer. *Clin Cancer Res* 2005;11:6650–6.

30. Imamura Y, Lochhead P, Yamauchi M, Kuchiba A, Qian ZR, Liao X, Nishihara R, Jung S, Wu K, Nosho K, Wang YE, Peng S, et al. Analyses of clinicopathological, molecular, and prognostic associations of KRAS codon 61 and codon 146 mutations in colorectal cancer: cohort study and literature review. *Mol Cancer* 2014;13:135.

31. Ogino S, Kawasaki T, Kirkner GJ, Kraft P, Loda M, Fuchs CS. Evaluation of markers for CpG island methylator phenotype (CIMP) in colorectal cancer by a large population-based sample. *J Mol Diagn* 2007;9:305–14.

32. Van Guelpen B, Dahlin AM, Hultdin J, Eklöf V, Johansson I, Henriksson ML, Cullman I, Hallmans G, Palmqvist R. One-carbon metabolism and CpG island methylator phenotype status in incident colorectal cancer: a nested case-referent study. *Cancer Causes Control* 2010;21:557–66.

33. Weisenberger DJ, Levine AJ, Long TI, Buchanan DD, Walters R, Clendenning M, Rosty C, Joshi AD, Stern MC, LeMarchand L, Lindor NM, Daftary D, et al. Association of the colorectal CpG island methylator phenotype with molecular features, risk factors, and family history. *Cancer Epidemiol Biomarkers Prev* 2015;24:512–9.

34. Weisenberger DJ, Siegmund KD, Campan M, Young J, Long TI, Faasse MA, Kang GH, Widschwendter M, Weener D, Buchanan D, Koh H, Simms L, et al. CpG island methylator phenotype underlies sporadic microsatellite instability and is tightly associated with BRAF mutation in colorectal cancer. *Nat Genet* 2006;38:787–93.

35. English DR, Young JP, Simpson JA, Jenkins MA, Southey MC, Walsh MD, Buchanan DD, Barker MA, Haydon AM, Royce SG, Roberts A, Parry S, et al. Ethnicity and risk for colorectal cancers showing somatic BRAF V600E mutation or CpG island methylator phenotype. *Cancer Epidemiol Biomarkers Prev* 2008;17:1774–80.

36. Eads CA, Danenberg KD, Kawakami K, Saltz LB, Blake C, Shibata D, Danenberg PV, Laird PW. MethyLight: a high-throughput assay to measure DNA methylation. *Nucleic Acids Res* 2000;28:E32.

37. Carr PR, Jansen L, Bienert S, Roth W, Herpel E, Kloor M, Bläker H, Chang-Claude J, Brenner H, Hoffmeister M. Associations of red and processed meat intake with major molecular pathological features of colorectal cancer. *Eur J Epidemiol* 2017;32:409–18.

38. Warth A, Kloor M, Schirmacher P, Bläker H. Genetics and epigenetics of small bowel adenocarcinoma: the interactions of CIN, MSI, and CIMP. *Mod Pathol* 2011;24:564–70.

39. Jass JR. Classification of colorectal cancer based on correlation of clinical, morphological and molecular features. *Histopathology* 2007;50:113–30.

40. Phipps AI, Limburg PJ, Baron JA, Burnett-Hartman AN, Weisenberger DJ, Laird PW, Sinicrope FA, Rosty C, Buchanan DD, Potter JD, Newcomb PA. Association between molecular subtypes of colorectal cancer and patient survival. *Gastroenterology* 2015;148:77-87.e2.

41. McCarthy AJ, Serra S, Chetty R. Traditional serrated adenoma: an overview of pathology and emphasis on molecular pathogenesis. *BMJ Open Gastroenterol* 2019;6:e000317.
